# Evaluation of the diagnostic value of magnetic resonance imaging combined with ultrasound and mammography for breast cancer using array spatial sensitivity encoding technique

**DOI:** 10.3389/fonc.2025.1596803

**Published:** 2025-11-03

**Authors:** Yingyi Xia, Zhiyu Ling, Hao Chen

**Affiliations:** ^1^ Department of Radiology, Yongkang First People’s Hospital, Yongkang, China; ^2^ Medical Department, Yongkang First People’s Hospital, Yongkang, China

**Keywords:** array spatial sensitivity encoding technique, magnetic resonance imaging, ultrasound, mammography, breast cancer

## Abstract

**Objective:**

This study aimed to evaluate the diagnostic efficacy of magnetic resonance imaging (MRI) combined with ultrasound and mammography for breast cancer (BC) using array spatial sensitivity encoding technique (ASSET).

**Methods:**

MRI images are processed using parallel imaging (PI) and ASSET techniques. The signal-to-noise ratio (SNR) of ASSET-diffusion-weighted imaging (DWI) and PI-DWI, as well as the contrast-to-noise ratio (CNR) between lesions and normal breast tissue, were compared. Image quality was also assessed. Using 70 cases of BC as the observation group (OG) and 70 non-BC cases as the control group (CG), the imaging characteristics of MRI, ultrasound, and mammography in both groups were compared. The Accuracy (*Acc*), Sensitivity (*Sen*), Specificity (*Spe*), and consistency of single and combined diagnosis using the three methodologies were evaluated.

**Results:**

Relative to the PI-DWI sequence, the ASSET-DWI sequence demonstrated notably shorter scanning time, higher CNR between lesions and normal breast tissue, better lesion visualization, clearer lesion margins, fewer image artifacts, and higher overall image quality (*P* < 0.05). In contrast to the CG, patients in the OG exhibited a higher proportion of irregular lesion morphology, non-smooth margins, and uneven enhancement on MRI, as well as a higher proportion of low echoic lesions, unclear boundaries, irregular morphology, irregular margins, posterior echo attenuation, and visible blood flow signals on ultrasound. Additionally, a higher proportion of irregular tumor margins, irregular morphology, spiculated signs, calcifications, and absence of capsule were observed on mammography (*P* < 0.05). Relative to MRI, ultrasound, and mammography alone, the combined diagnostic method showed significantly higher *Acc*, *Sen*, *Spe*, and Kappa values (*P* < 0.05).

**Conclusion:**

The combined use of MRI, ultrasound, and mammography based on ASSET for BC diagnosis offers significant advantages, providing clinicians with more reliable diagnostic tools.

## 1 Introduction

In the current medical field, breast cancer (BC), as one of the most common malignant tumors among women, early diagnosis is crucial for improving treatment outcomes and survival rates. With the development of society and the increasing awareness of health, there is a growing demand for early detection and accurate diagnosis of BC (1). In this context, the continuous advancement of medical imaging technology has provided powerful support for addressing this issue.

Magnetic resonance imaging (MRI), ultrasound examination, and mammography, among other imaging techniques, have been widely used in the diagnosis of BC due to their high sensitivity (*Sen*) and resolution (2–4). MRI is a non-invasive imaging technique that produces high-contrast three-dimensional images, providing detailed anatomical information to physicians. Its *Sen* to signal intensity and contrast enables clear visualization of abnormalities within breast tissue, including the size, shape, location, and relationship to surrounding tissues of tumors, thus providing robust support for early detection of BC (5). Ultrasound examination, on the other hand, is a real-time, radiation-free imaging technique that plays a crucial role in BC diagnosis (6). By reflecting and propagating ultrasound waves, it generates dynamic images of breast tissue, capturing real-time changes in tissue structure. This is vital for distinguishing between fluid and solid tissues, observing blood flow conditions, and detecting abnormal morphological features. In cases of dense breast tissue, ultrasound examination provides intuitive and reliable information, offering physicians a more comprehensive screening tool for BC (7). Mammography plays a unique role in breast X-ray imaging, particularly excelling in the detection of small calcifications (8). It provides clear visualization of the morphology and distribution of calcifications, which is crucial for assessing abnormalities in breast tissue. Small calcifications are often indicative of early BC, thus mammography plays a key role in the early screening and diagnosis of BC (9). However, individual imaging techniques also have inherent limitations, restricting their comprehensive application in BC diagnosis, particularly MRI.

MRI primarily generates high-contrast images by detecting signals from water molecules in the human tissue, making it susceptible to motion artifacts during imaging. Motion artifacts typically occur when patients move or breathe during MRI scans, leading to image blurring, distortion, or inaccurate structures (10, 11). This is particularly crucial for breast MRI as breast tissue itself may be subject to physiological motion such as respiration and cardiac pulsation during scanning. These motion-induced artifacts may obscure or blur potential lesions, affecting the accurate detection and localization of conditions like BC. To mitigate the impact of motion artifacts in MRI, a common approach is to employ fast imaging techniques such as fast gradient echo (FGRE) or spiral scanning to shorten scan times and reduce patient motion during imaging (12, 13). Parallel imaging (PI) (14) and array spatial sensitivity encoding technique (ASSET) (15) are combined to further enhance MRI image quality and acquisition efficiency. ASSET, as an optimized implementation of PI, is employed to reconstruct images by exploiting the spatial *Sen* information of receiver coils, which not only accelerates scanning but also improves spatial resolution and diagnostic stability. Recent studies have shown that multimodal imaging combined with ASSET has high potential in BC detection. Leung et al. (2024) (16) reported in a systematic Meta-analysis that the ASSET-assisted detection method integrating multi-source imaging information significantly improved the accuracy (*Acc*) and consistency of BC detection, especially in identifying complex anatomical structures and early-stage micro-lesions. Nevertheless, systematic integration of MRI (especially ASSET-based MRI), ultrasound, and mammographic images into a clinically applicable comprehensive assessment framework remains largely unexplored.

Therefore, this study is designed to evaluate the application value of MRI based on PI and ASSET technology, combined with ultrasound and mammography, in BC diagnosis. A multimodal complementary mechanism and comprehensive evaluation index system were established, and the *Acc* and feasibility of the method were comprehensively assessed, so that the level of early BC diagnosis could be improved and more timely and effective therapeutic strategies could be provided for patients.

## 2 Research methodologies

### 2.1 Research object

This study recruited 70 patients diagnosed with malignant BC through pathological examination at the First People’s Hospital of Yongkang City from January 2020 to December 2023, referred to as the observation group (OG). Upon enrollment, these patients underwent a series of relevant examinations, including MRI, ultrasound, and mammography, with a final diagnosis confirmed by pathological examination. All 70 patients exhibited prominent symptoms of BC at the time of enrollment, primarily including palpable masses, breast swelling, and nipple retraction. The age range of the study subjects was 26 to 90 years, with a mean age of 52.77 ± 12.46 years; 34 cases (48.57%) had lesions on the left side, 36 cases (51.43%) on the right side, and none had bilateral involvement (0%). To control potential selection bias, all cases were enrolled consecutively and were screened according to preset inclusion and exclusion criteria, so that complete clinical data and full examination profiles were ensured. In addition, to minimize confounding, 70 patients who underwent breast surgery or core-needle biopsy during the same period and whose pathological diagnosis was non-BC (benign lesions) were selected as the control group (CG) after the OG had been enrolled; these controls were matched 1:1 by age, with an allowable error of ±5 years within each stratification, so that comparability was strengthened. The age range of patients in the CG was 20-90 years, and the mean age was recorded as 43.21 ± 10.68 years; 32 cases (45.71%) had lesions on the left side, 32 cases (45.71%) on the right side, and 6 cases (8.57%) had bilateral involvement. No statistically significant differences were observed between the two groups in age, lesion laterality, or other general characteristics (*P*>0.05), indicating that the groups were comparable. The specific inclusion and exclusion criteria are as follows:

Inclusion criteria: A: All patients in the OG were pathologically confirmed by surgical or core-needle biopsy specimens to have common-type BC (inflammatory BC, medullary carcinoma, papillary carcinoma, and other special subtypes were excluded); B: all patients in the CG were pathologically verified to have benign, non-BC lesions; C: all participants were willing and able to complete every required study procedure; D: all patients had no other complications; E: all patients did not have severe systemic diseases; F: all patients had not received treatment for BC; G: all patients had no history of breast surgery or breast inflammation; H: all patients were not pregnant or lactating; I: all patients had no history of allergy to contrast agents used in CT or MRI examinations.
Exclusion criteria: A: patients with concomitant other types of malignant tumors; B: patients with severe heart disease, kidney disease, or other major organ dysfunction; C: patients with mental illness or cognitive impairment; D: patients who could not understand or comply with the study requirements; E: patients with contraindications to MRI examinations, such as pacemakers or metal implants.

### 2.2 Test methodologies

In this study, all patients underwent MRI, ultrasound, and mammography of the breast.

#### 2.2.1 MRI

##### 2.2.1.1 Image acquisition

Breast MRI examinations were conducted using a 1.5T HDX ECHOSPEED 8-CH MRI system from GE Healthcare and a 1.436T U586 MRI system from United Imaging. The specific procedures were as follows:

- The examination was scheduled on the 8th day after the completion of menstruation.
- The patients were placed in the prone position, and sandbags or abdominal belts were applied to the abdomen prior to examination to reduce motion artifacts caused by respiratory motion.
- GE used an 8-channel breast-specific coil, while United Imaging employed a 10-channel breast-specific coil to ensure high clarity and *Acc* in breast imaging.
- The acquired scanning sequences included: axial short T1 inversion recovery (AX STIR), axial T1-weighted imaging (AX T1), axial diffusion-weighted imaging (AX DWI), sagittal STIR (SAG STIR), axial 3D dynamic T1-weighted imaging (AX 3D T1dyn), and sagittal T1 contrast-enhanced imaging (SAG T1+C).
- Dynamic contrast-enhanced scanning was performed using gadolinium-diethylene triamine pentaacetic acid (Gd-DTPA) contrast agent (dose: 0.2 mmol/kg), with rapid dynamic enhancement scanning immediately after contrast agent injection. The United Imaging axial DWI sequence employs the PI processing technology, while the GE 1.5T uses the ASSET processing technology. The scanning parameters for the United Imaging U586 and GE 1.5T axial STIR scans are as follows:

United Imaging slice thickness: 4 mm; gap: 0.8mm; field of view (FOV): 300×300; matrix size: 336×89; TR: 4376 ms; TE: 30.96 ms; excitation flip angle: 90°; refocusing flip angle: 150°; average: 1.8; bandwidth: 300 Hz/pixel; inversion time: 175 ms; echo train length: 7.
GE1.5: slice thickness: 5.5mm; gap: 1mm; FOV: 330×330; matrix size: 320×192; TR: 6600 ms; TE: 42 ms; average: 2; bandwidth: 41.67 Hz/pixel; inversion time: 145 ms; echo train length: 14.

F. Special attention was paid to the breast mass area, and dynamic signal intensity data were obtained by delineating the enhancement regions of the contralateral breast.
G. Three-dimensional reconstruction analysis of tumor morphology and characteristics was performed using the acquired data.

##### 2.2.1.2 Image processing under ASSET and PI technologies

The image post-processing was performed separately on the uWSMR workstation (United Imaging) and the ADW4.4 workstation (GE Healthcare). Regions of interest (ROIs) were delineated on the transverse ASSET-DWI and PI-DWI images, and the signal intensity (SI) and standard deviation (SD) of breast tumor lesions (SI _lesions_ and SD _lesions_) were measured, as well as the SI and SD of adjacent normal breast tissues (SI _normal breast_ and SD _normal breast_). Simultaneously, the noise (SD _background_) of background tissue was measured. Signal-to-noise ratios (SNR _lesion_ and SNR _normal breast_) and contrast-to-noise ratios (CNR _lesion-normal breast_) between lesions and normal breast tissue were calculated for both sets of images. When delineating ROIs, areas without artifacts or deformities were selected, and care was taken to avoid edematous, necrotic, and hemorrhagic regions.

##### 2.2.1.3 Image quality assessment

Two experienced radiologists from the MRI department independently performed blinded image reading and evaluated the image quality of the ASSET-DWI and PI-DWI image sets. A 5-point Likert scale was used to assess parameters including lesion visibility, lesion edge definition, image artifacts, and overall image quality (17), with detailed scoring criteria provided in **Table 1**. During the blinded evaluation, both assessors independently scored each image set without prior knowledge of clinical information, ensuring independence and impartiality. Each assessor based their scores solely on image quality, without referencing any clinical data or patient history. To avoid subjective bias, assessors were not shown the results or scores of the other evaluator and the image sequence was randomized to ensure independent judgment for each image. In cases where significant discrepancies in scoring were found between the two evaluators, the research team would discuss the cases and revise the final score based on a consensus.

**Table 1:** Scoring standards for each indicator.

Score	Lesion visibility	Edge sharpness	Artifacts	Overall quality
1 point	Not visible	Not recognizable	Severe, cannot diagnose	Poor, can’t diagnose
2 points	Faintly visible	Extremely blurry	Severe, affecting local area	Poor, diagnosis difficult
3 points	Blurred but recognizable	Blurred but recognizable	Moderate, minor impact	Fair, diagnosis possible
4 points	Moderately clear	Adequate	Slight, does not affect diagnosis	Good
5 points	Clear	Clear	Clear, none	

#### 2.2.2 Ultrasound examination

The ADM color Doppler ultrasound diagnostic instrument (GE Healthcare) was utilized, with a probe frequency range of 7~12MHz. This equipment was employed for two-dimensional ultrasound examination of breast nodules, to observe various aspects including the location, morphology, size, margins, internal echoes, ductal dilation, and lymph node enlargement of the nodules. During the examination, the CDFI mode could be switched on to detect blood flow signals inside and around the nodules, evaluating the blood flow condition of the lesions, and detecting parameters such as blood flow resistance index and spectrum. Subsequently, the UE mode could be activated, with the selected ROI being approximately 2 to 3 times the area of the lesion. The probe was positioned perpendicular to the skin, lightly touching the lesion area, and applying slight pressure to achieve a frequency of around 2.5 MHz. In this mode, images could be frozen and saved, while simultaneously observing the imaging characteristics of the lesion tissue and surrounding tissues in real time. All ultrasound images were independently evaluated by two physicians from the ultrasound department, who assessed the lesion characteristics under different imaging modes. The evaluators independently scored indicators such as the echogenicity pattern, border features, morphology, and blood flow of the nodules, and made benign or malignant diagnoses based on the breast imaging reporting and data system (BI-RADS) classification standard. During the evaluation, the image data were presented randomly, without referencing any patient clinical information. In cases of disagreement, the two physicians reached a consensus through discussion.

#### 2.2.3 Molybdenum-targeted X-ray

All patients underwent mammography using X-ray. The equipment included a Computed Radiography (CR) system (IMS, Model: GIOTTO) and a Direct Digital Radiography (DR) system (Shengnuo, Model: Navigator Mammography DR/SN-DR3). All examinations were performed using digital imaging methods. During the examination, patients were required to undergo bilateral breast imaging in both the craniocaudal and mediolateral oblique positions to comprehensively assess breast tissue. Depending on the specific condition of the patient, linear or local magnification imaging of the lesion area might be performed if necessary to observe the details of the lesion more comprehensively. The examination was conducted in fully automated mode, with precise pressure control applied during breast compression, typically maintained between 7 to 12 N. During X-ray mammography, healthcare professionals recorded the shape, size, margin features, and any calcifications of the mass. These records are crucial for determining the presence of abnormalities or potential lesions in breast tissue and understanding their nature and extent. All mammographic X-ray images were independently evaluated by two radiologists specializing in mammography, with the evaluators having no access to patient information or prior diagnostic results. Each evaluator independently assessed lesion characteristics in the images, such as shape, margin, size, and calcifications, according to the standard BI-RADS scoring system, and made a benign or malignant diagnosis. To minimize bias, the evaluation process was conducted separately, and in cases of disagreement, consensus was reached through discussion.

#### 2.2.4 Comprehensive imaging evaluation

**Figure 1:**
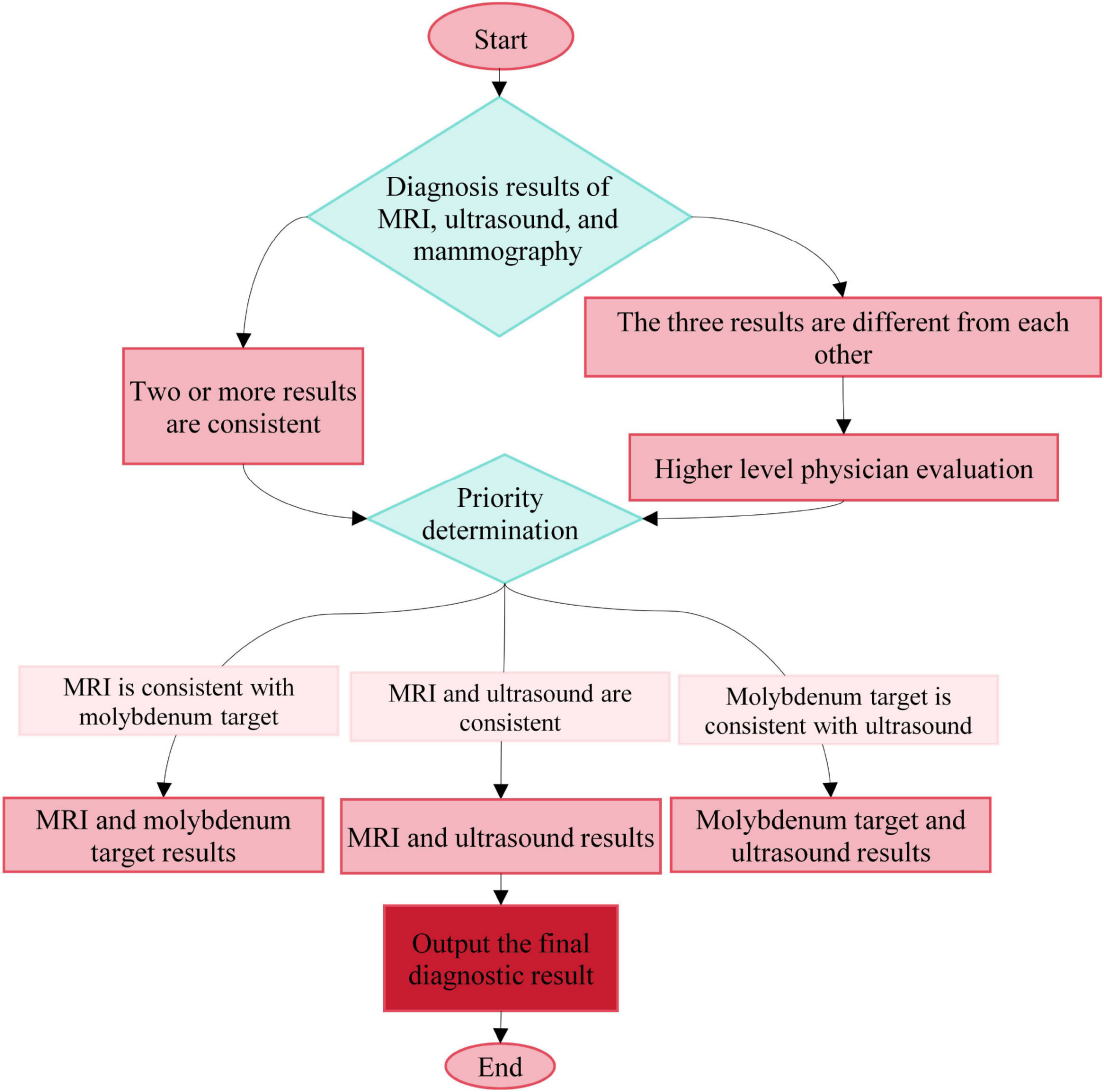
Combined diagnostic decision-making process.

In this study, the value of combined MRI, ultrasound, and mammography was further evaluated. Images from MRI, ultrasound, and mammography were independently assessed by two experienced radiologists with intermediate or higher professional titles from the respective departments, and strict blinding was applied; the evaluators were kept unaware of the results from the other modalities to ensure independence. To control subjective error and enhance consistency, all physicians were uniformly trained, and clear criteria for BI-RADS classification and interpretation standards in each imaging modality were established. Image data were presented in random order, and inter- and intra-observer agreement was analyzed by Kappa statistics; any diagnostic disagreement was resolved through discussion between the two physicians and, when necessary, by referral to a third senior physician. For combined diagnosis, the rule was applied that if two or more of the three imaging methods yielded concordant results, the corresponding diagnosis was adopted as the final primary diagnosis. The specific decision-making logic is illustrated in **Figure 1**. These measures effectively improved the standardization and consistency of multimodal image interpretation and provided a safeguard for the *Acc* of the combined diagnostic approach.

### 2.3 Observation indexes

1. The image acquisition time was recorded, and the SNR _lesion_, SNR _normal breast_, and CNR _lesion-normal breast_ between lesions and normal breast tissue were observed and compared for both sets of MRI images: ASSET-DWI and PI-DWI.
2. The performance of MRI images, ultrasound images, and molybdenum-targeted X-ray images of lesions was compared between the CG and the OG. Morphology, margins, and enhancement were assessed for MRI images; echogenicity, borders, morphology, margins, posterior acoustic features, and blood flow signals within the lesion were assessed for ultrasound images; edges, morphology, lobulation, spiculation, and calcification of the masses were assessed for molybdenum-targeted X-ray images.
3. Pathological examination results were used as the gold standard for final diagnosis. The diagnostic performance of ASSET-based MRI, ultrasound, molybdenum-targeted X-ray, and the combination of the three methodologies for BC diagnosis, including *Acc*, *Sen*, and Specificity (*Spe*), was compared as shown in Equations 1–3.

(1) Acc=(TP+TN)/(TP+TN+FP+FN)×100%

(2) Sen=TP/(TP+FN)×100%

(3) Spe=TN/(TN+FP)×100%

Among them, *TP* represents True Positives, *TN* represents True Negatives, *FP* represents False Positives, and *FN* represents False Negatives.

### 2.4 Statistical methodologies

Statistical analysis of the acquisition time for ASSET-DWI and PI-DWI, as well as the comparison of SNR and CNR between the two image sets, was conducted using SPSS 26.0 statistical software. Firstly, a normality test was performed. If the data followed a normal distribution, a paired sample t-test was used for intergroup comparison; otherwise, the Wilcoxon signed-rank test was employed. The intergroup comparison of subjective image quality scores was also conducted using the Wilcoxon signed-rank test. Differences were considered statistically significant at a level of *P* < 0.05. Additionally, Kappa test was employed to evaluate the consistency between pathological diagnosis results and the joint diagnosis based on ASSET using MRI, ultrasound, mammography, and the combination of the three methodologies. The interpretation of Kappa values is as follows: 0 to 0.20 indicates inconsistency, 0.21 to 0.40 indicates poor consistency, 0.41 to 0.60 indicates moderate consistency, 0.61 to 0.80 indicates good consistency, and 0.81 to 1.00 indicates excellent consistency.

## 3 Results

### 3.1 Evaluation of image acquisition time, SNR, and CNR of ASSET-DWI and PI-DWI

**Figure 2:**
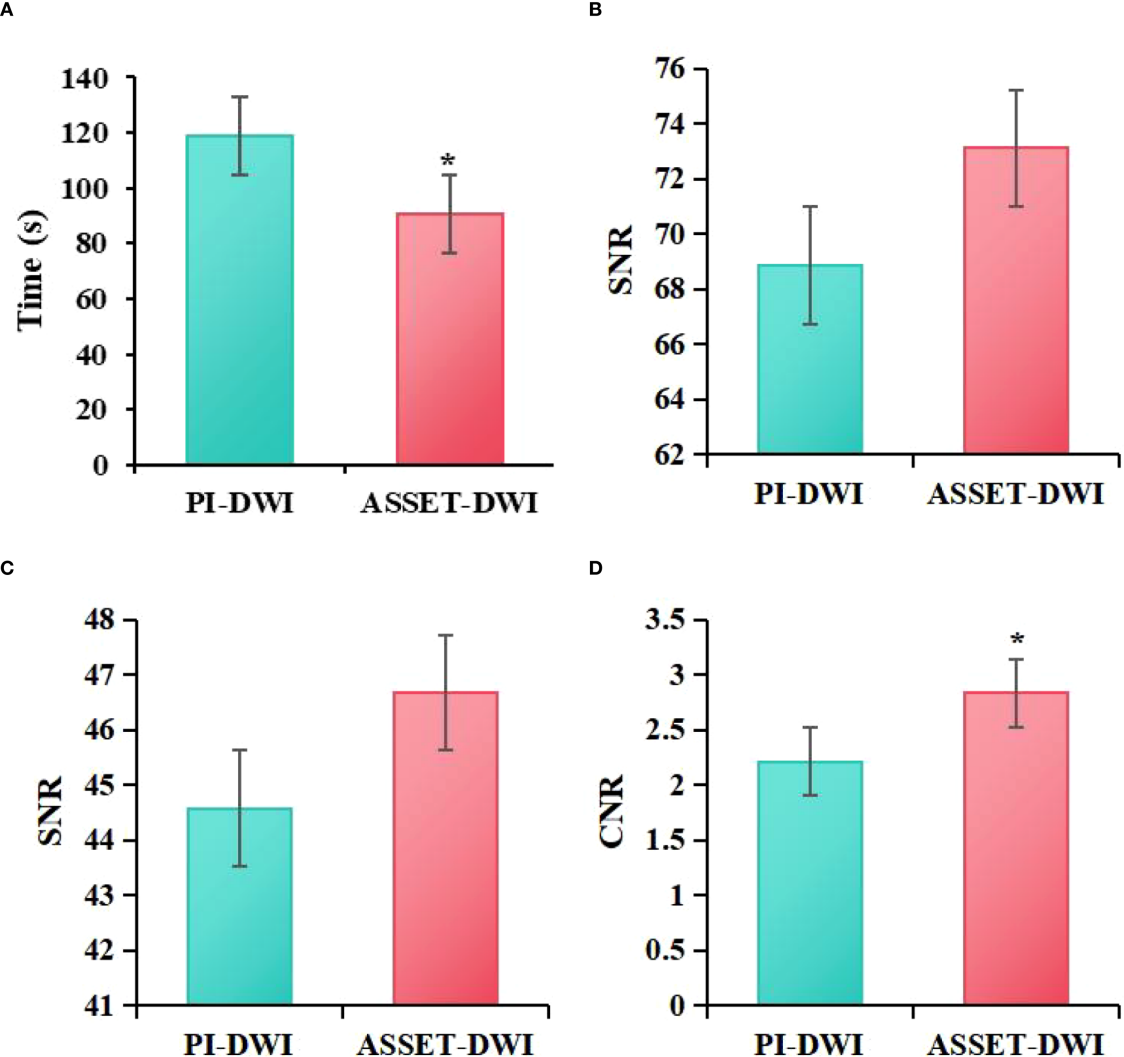
Comparison of image acquisition time **(A)**, SNR of lesions **(B)**, SNR of normal breast tissue **(C)**, and CNR of lesions to normal breast tissue **(D)** between ASSET-DWI and PI-DWI images (*P<0.05 vs. PI-DWI).

The acquisition times of PI-DWI and ASSET-DWI images were recorded in the study, revealing that the acquisition time for PI-DWI was (118.92 ± 9.03) s, while that for ASSET-DWI images was (102.00 ± 6.21) s. The SNR of lesions, SNR of normal breast tissue, and CNR of lesions to normal breast tissue for PI-DWI images were (68.87 ± 49.34), (44.58 ± 28.67), and (2.21 ± 1.21), respectively, while for ASSET-DWI images, they were (73.11 ± 45.95), (46.67 ± 30.13), and (2.83 ± 1.67), respectively. Through statistical analysis, it was found that in contrast to the PI-DWI sequence, the ASSET-DWI sequence exhibited greatly lower scan times (*P = 0.001*). The SNR of lesions and SNR of normal breast tissue differed slightly between PI-DWI and ASSET-DWI images (*P = 0.105*). However, the CNR of lesions to normal breast tissue in ASSET-DWI images was markedly superior to that in PI-DWI images (*P = 0.005*) (**Figure 2**).

### 3.2 ASSET-DWI and PI-DWI image quality scoring

**Figure 3:**
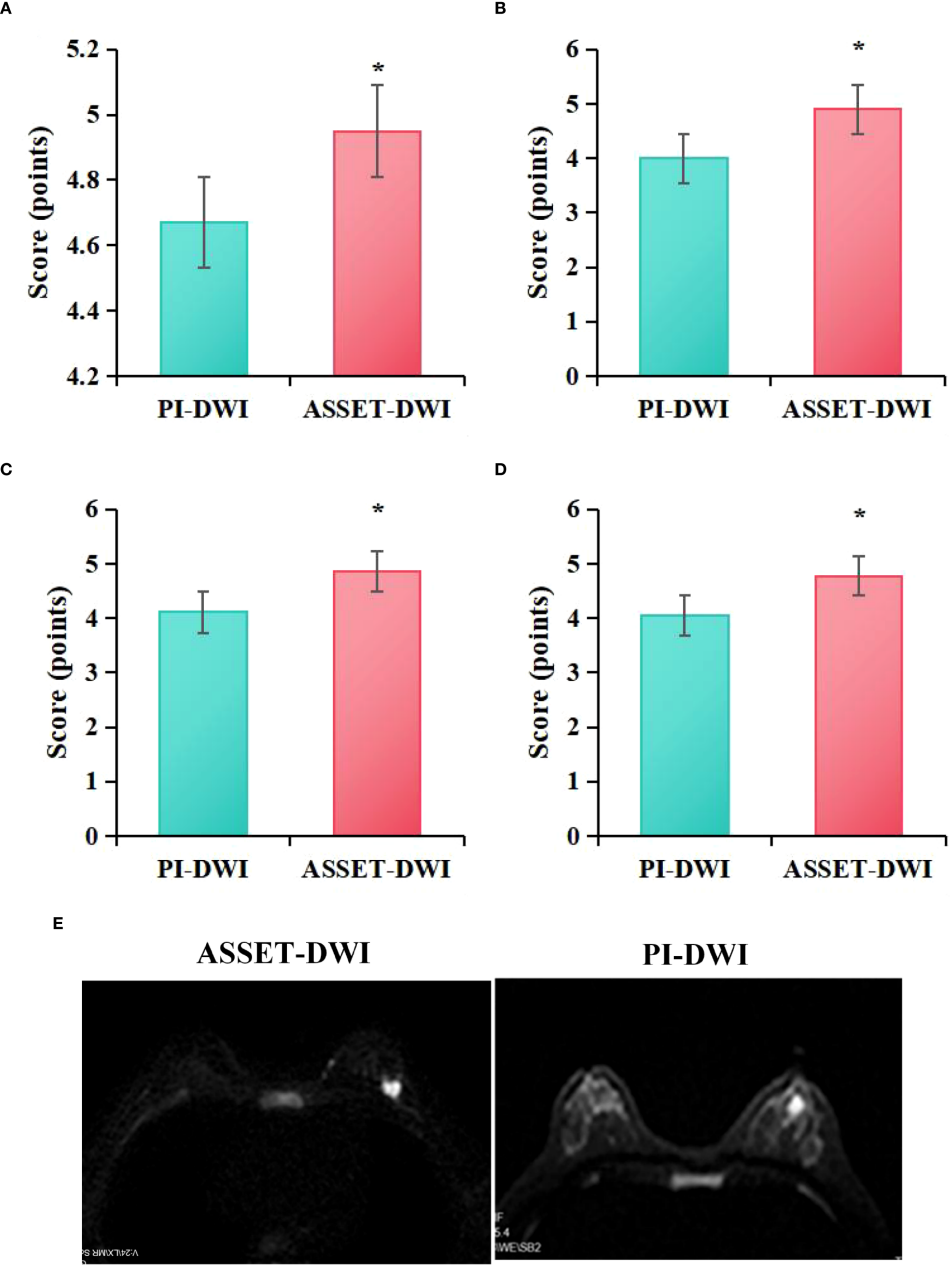
Quality assessment scores of ASSET-DWI and PI-DWI images **(A)** lesion visibility, **(B)** lesion edge sharpness, **(C)** artifacts, **(D)** overall quality; **(E)** MRI image contrast; *P<0.05 vs. PI-DWI.

**Figure 3** presents the evaluation of image quality between PI-DWI and ASSET-DWI. Specifically, the lesion visibility score for PI-DWI images was (4.67 ± 0.37), lesion edge sharpness score was (4.00 ± 0.32), artifact score was (4.11 ± 0.56), and overall quality score was (4.05 ± 0.55). For ASSET-DWI images, the lesion visibility score was (4.95 ± 0.32), lesion edge sharpness score was (4.90 ± 0.39), artifact score was (4.86 ± 0.58), and overall quality score was (4.77 ± 0.54). Statistical analysis demonstrated that, compared to PI-DWI images, ASSET-DWI images received significantly higher scores in lesion visibility (*P* = 0.021), lesion edge sharpness (*P* = 0.011), image artifacts (*P* = 0.033), and overall image quality (*P* = 0.003).

### 3.3 MRI image performance

**Figure 4:**
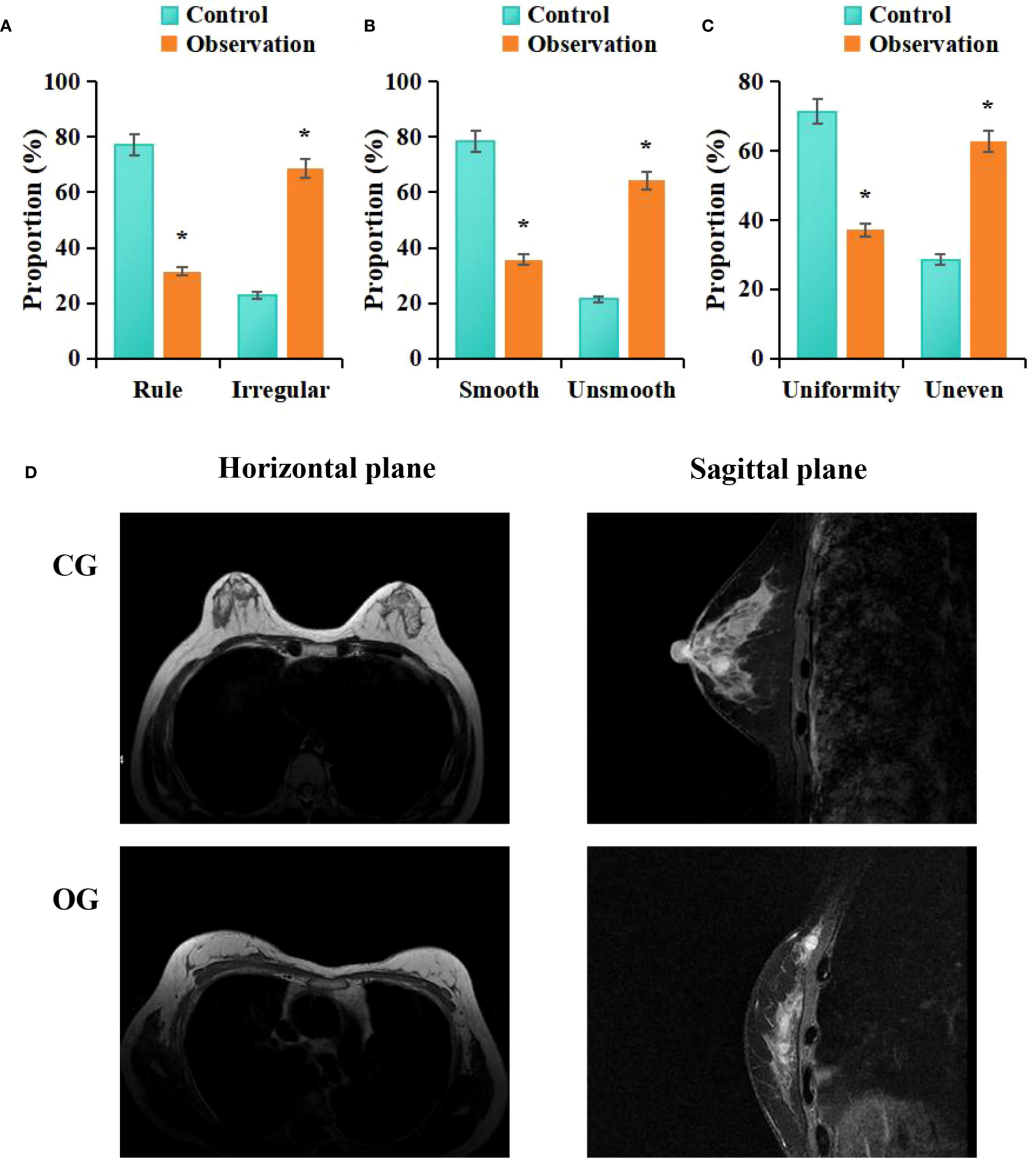
Comparison of MRI imaging features between groups **(A)** morphology, **(B)** margins, **(C)** enhancement; **(D)** MRI images; #*P* < 0.05 vs. CG.

According to the statistical analysis in this study, among the patients in the CG, 22 cases (31.43%) exhibited irregular morphology in MRI images, while 48 cases (68.57%) exhibited regular morphology; 55 cases (64.28%) had smooth margins, whereas 25 cases (35.71%) had irregular margins; and 44 cases (62.86%) showed homogeneous enhancement, while 26 cases (37.14%) showed heterogeneous enhancement. In contrast, among the patients in the OG, 54 cases (31.43%) exhibited irregular morphology, whereas 16 cases (22.86%) exhibited regular morphology; 15 cases (21.43%) had smooth margins, while 55 cases (78.57%) had irregular margins; and 20 cases (28.57%) showed homogeneous enhancement, while 50 cases (71.43%) showed heterogeneous enhancement. Statistical analysis revealed that, compared to the CG, the OG exhibited a higher proportion of patients with irregular shape (*P* = 0.002), irregular margins (*P* = 0.001), and heterogeneous enhancement (*P* = 0.009) (*P* < 0.05) (**Figure 4**).

### 3.4 Ultrasonic image representation

**Figure 5:**
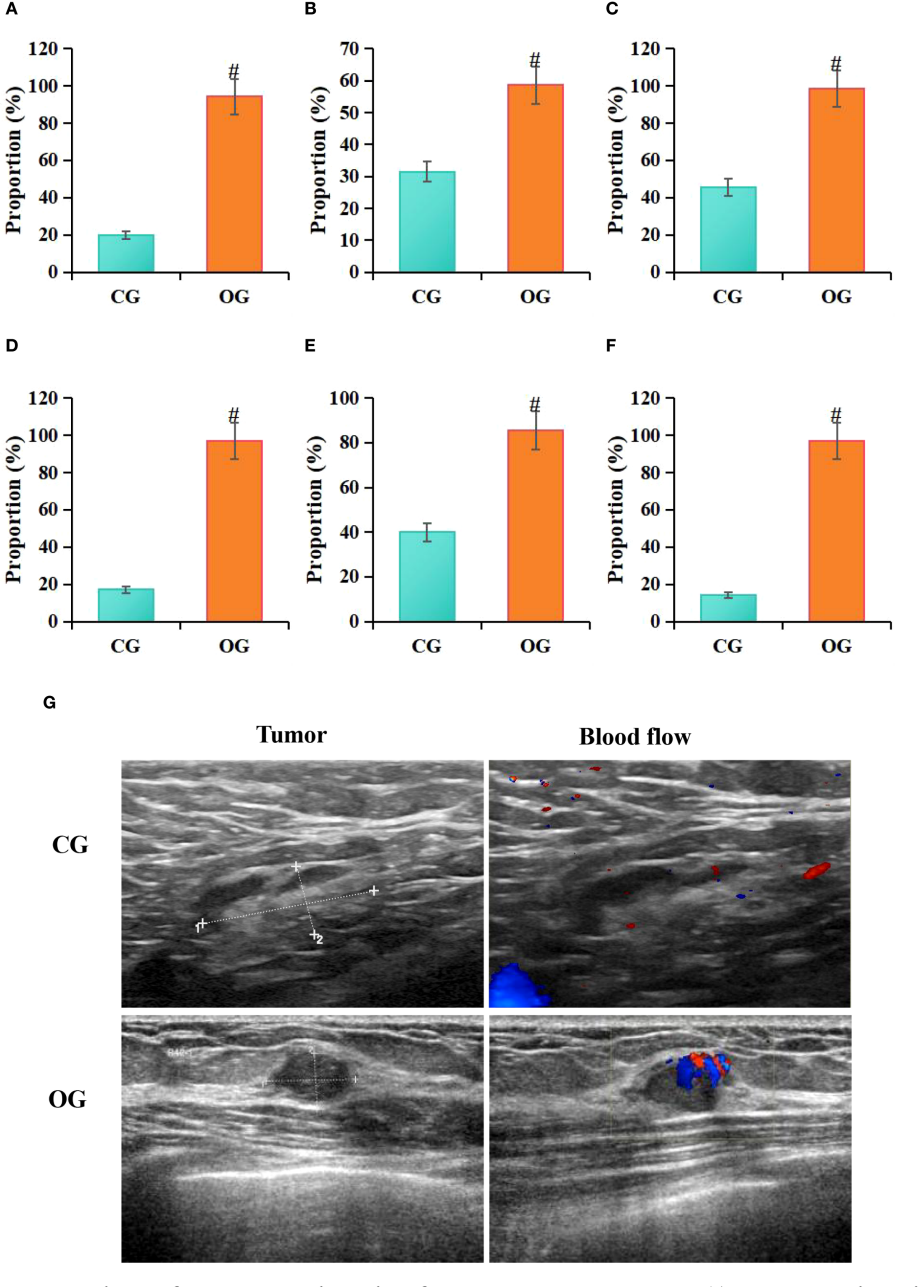
Comparison of ultrasound imaging features between groups **(A)** hypoechoic lesions, **(B)** indistinct margins, **(C)** irregular shape, **(D)** irregular margins, **(E)** posterior acoustic attenuation, **(F)** longitudinal-to-transverse ratio>1; **(G)** ultrasound images; #*P*<0.05 vs. CG.

According to statistical analysis in this study, among patients in the CG, 14 cases (20%) exhibited hypoechoic lesions, 22 cases (31.43%) had indistinct lesion borders, 32 cases (45.71%) showed irregular morphology, 12 cases (17.14%) had irregular lesion edges, 28 cases (40%) showed posterior acoustic attenuation, and 10 cases (14.29%) exhibited visible blood flow signals on ultrasound images. In contrast, among patients in the OG, 66 cases (94.29%) exhibited hypoechoic lesions, 41 cases (58.57%) had indistinct lesion borders, 69 cases (98.57%) showed irregular morphology, 68 cases (98.14%) had irregular lesion edges, 60 cases (85.71%) exhibited posterior acoustic attenuation, and 68 cases (97.14%) showed visible blood flow signals on ultrasound images. Statistical analysis showed that, compared to the CG, the OG had a higher proportion of patients with hypoechoic lesions (*P* = 0.026), unclear lesion borders (*P* = 0.039), irregular shape (*P* = 0.042), irregular lesion margins (*P* = 0.009), posterior acoustic attenuation (*P* = 0.006), and visible blood flow signals (*P* = 0.009) (P < 0.05) (**Figure 5**).

### 3.5 X-ray manifestation of molybdenum-targeted

**Figure 6:**
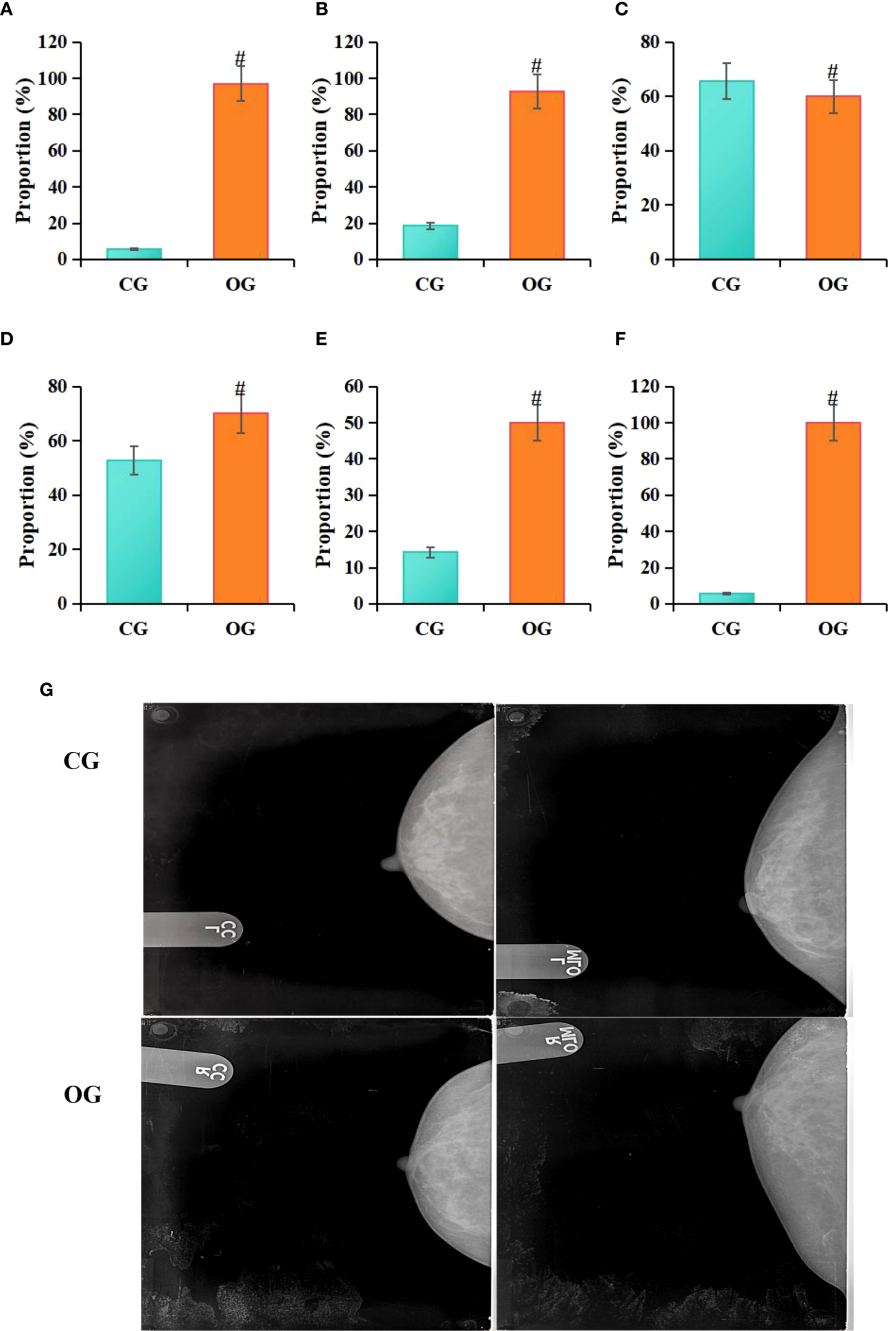
Comparison of molybdenum-targeted X-ray findings between groups **(A)** irregular margins, **(B)** irregular shape, **(C)** lobulated margin, **(D)** spiculated margin, **(E)** calcifications, **(F)** lack of capsule; **(G)** molybdenum target X-ray image; RCC, right breast craniocaudal view; RMLO, right breast mediolateral oblique view; LCC, left breast craniocaudal view; LMLO, left breast mediolateral oblique view; #*P*<0.05 vs. CG.

Statistical analysis conducted in this study revealed that among patients in the CG, 4 cases (5.71%) exhibited irregular tumor margins on molybdenum-targeted X-ray images, 13 cases (18.57%) showed irregular tumor morphology, 46 cases (65.71%) exhibited lobulated tumor margins, 37 cases (52.86%) showed spiculated tumor margins, 10 cases (14.29%) exhibited calcifications, and 4 cases (5.71%) had no capsule. In contrast, among patients in the OG, 68 cases (97.14%) exhibited irregular tumor margins, 65 cases (92.86%) showed irregular tumor morphology, 42 cases (60%) exhibited lobulated tumor margins, 49 cases (70%) showed spiculated tumor margins, 35 cases (50%) exhibited calcifications, and 70 cases (100%) had no capsule on molybdenum-targeted X-ray images. Statistical analysis indicated that, compared to the CG, the OG had a higher proportion of patients with irregular tumor margins (*P* = 0.012), irregular tumor shape (*P* = 0.011), radial tumor margins (*P* = 0.002), calcifications (*P* = 0.007), and absence of a capsule (*P* = 0.027) (**Figure 6**).

### 3.6 Statistics of MRI, ultrasound, molybdenum-targeted X-ray, combined method, and pathological examination results

In the study, a positive result was defined as the diagnosis of BC, while a negative result was defined as the absence of BC. According to the statistical analysis conducted in this study, among 140 patients, 70 cases were diagnosed with negative pathology results and 70 cases with positive pathology results, accounting for 50% each. For MRI diagnosis, 74 cases (52.85%) were negative and 66 cases (47.14%) were positive. For ultrasound diagnosis, 73 cases (52.14%) were negative and 67 cases (47.86%) were positive. For molybdenum-targeted X-ray diagnosis, 69 cases (49.29%) were negative and 71 cases (50.71%) were positive. For combined diagnosis, 73 cases (52.14%) were negative and 67 cases (47.85%) were positive (**Table 2**).

**Table 2:** Statistics of MRI, ultrasound, molybdenum-targeted X-ray, combined method, and pathological examination results.

Diagnostic method	Pathological result (negative)	Pathological result (positive)	Total
MRI (Negative)	54	20	74
MRI (Positive)	16	50	66
Ultrasound (Negative)	45	28	73
Ultrasound (Positive)	25	42	67
Mammography (Negative)	49	20	69
Mammography (Positive)	21	50	71
Combination (Negative)	68	5	73
Combination (Positive)	2	65	67
Total	70	70	140

### 3.7 Comparison of diagnostic efficiency of MRI, ultrasound, molybdenum-targeted X-ray, and combined methodologies

**Figure 7:**
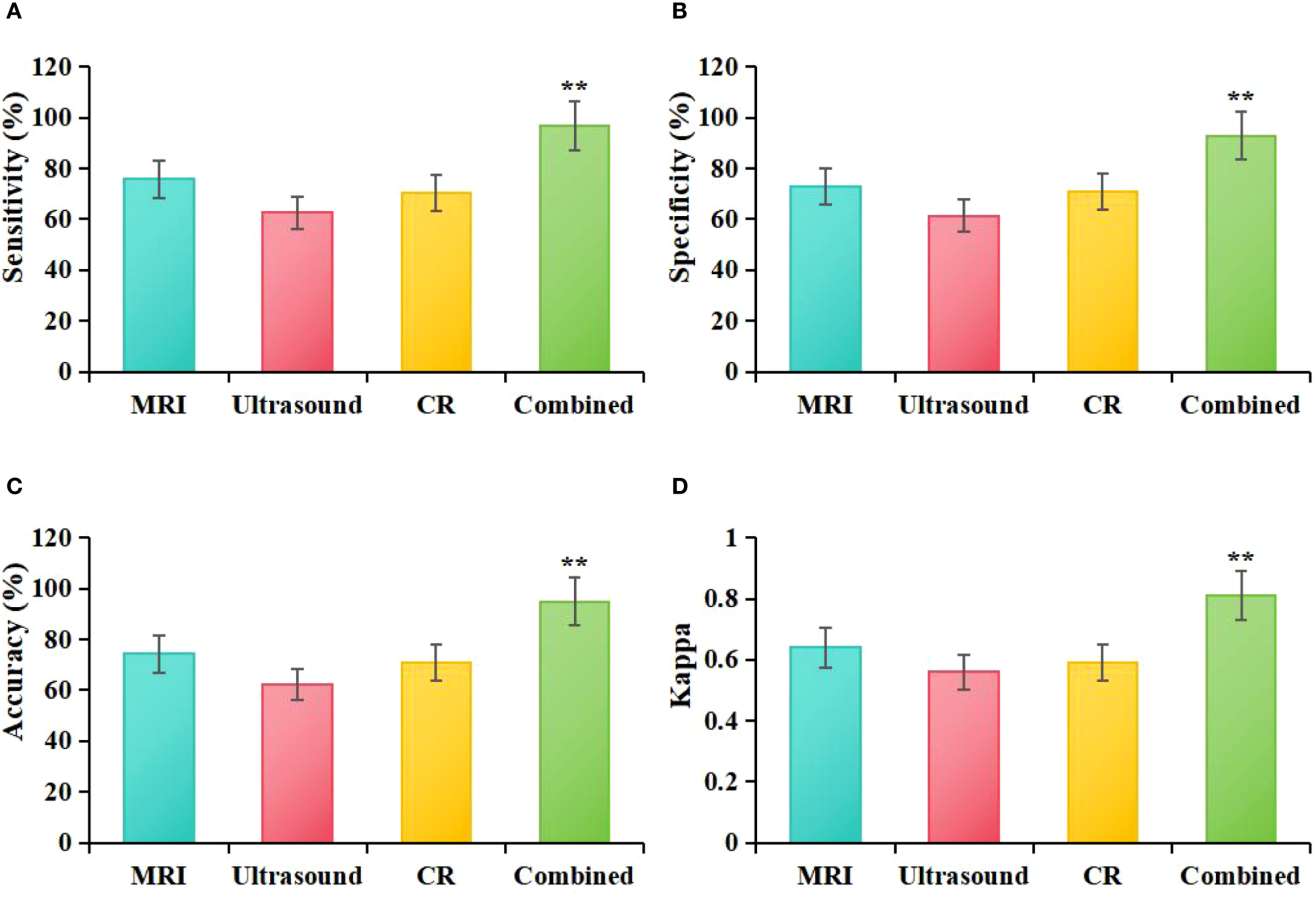
Comparative diagnostic efficacy of MRI, ultrasound, molybdenum-targeted X-ray, and combined methodologies **(A)**: *Sen*, **(B)**: *Spe*, **(C)**: *Acc*, **(D)**: Kappa; **P<0.05 vs. MRI, ultrasound, and molybdenum-targeted X-ray.

Based on the statistical results from **Table 2**, the study further calculated the diagnostic *Acc*, *Sen*, *Spe*, and agreement (Kappa) for MRI, ultrasound, molybdenum-targeted X-ray, and combined methodologies. The results showed that the diagnostic *Acc*, *Sen*, *Spe*, and Kappa value for MRI were 74.29%, 75.76%, 73.00%, and 0.64, respectively. For ultrasound, the corresponding values were 62.14%, 62.69%, 61.51%, and 0.56; for mammography, the values were 70.71%, 70.42%, 71.01%, and 0.59. The diagnostic *Acc*, *Sen*, *Spe*, and Kappa value for the combined diagnostic method were 95.00%, 97.01%, 93.01%, and 0.81, respectively. Statistical analysis showed that, compared to MRI based on ASSET technology, the combined diagnostic method significantly outperformed MRI in terms of *Acc* (*χ²=12.37, P = 0.003*), *Sen* (*χ²* = 14.22, *P* = 0.002), *Spe* (*χ²* = 11.85, *P* = 0.004), and Kappa (*Z* = 4.32, *P* = 0.005) values. When compared to ultrasound, the combined diagnostic method also demonstrated significantly higher *Acc* (*χ²* = 18.92, *P* = 0.001), *Sen* (*χ²* = 20.15, *P* = 0.001), *Spe* (*χ²* = 16.74, *P* = 0.002), and Kappa (*Z* = 5.67, *P* = 0.003) values. In comparison to mammography, the combined diagnostic method exhibited significantly superior *Acc* (*χ²* = 15.06, *P* = 0.002), *Sen* (*χ²* = 16.33, *P* = 0.003), *Spe* (*χ²* = 13.45, *P* = 0.004), and Kappa (*Z* = 4.89, *P* = 0.009) values. Furthermore, when compared, MRI based on ASSET technology showed higher diagnostic *Acc*, *Sen*, *Spe*, and Kappa values than both ultrasound and mammography (**Figure 7**).

## 4 Discussion

The aim of this study was to evaluate the effectiveness of combining ASSET -based MRI, ultrasound, and mammography in the diagnosis of BC. By integrating the strengths of these different imaging techniques, more comprehensive information can be provided, thereby enhancing the diagnostic *Acc* of BC.

Breast MRI provides high-resolution imaging that offers detailed views of breast tissue with high contrast, making it particularly useful for patients with dense breast tissue and cases that are difficult to detect using other diagnostic methods (18). Typical BC lesions on MRI appear as irregular or spiculated masses with poorly defined borders and exhibit rapid enhancement and washout characteristics in dynamic contrast-enhanced scans (5, 19). This study found that MRI findings in BC patients often present as irregular lesion shape, smooth edges, and heterogeneous enhancement. Color Doppler ultrasound (CDFI), as an important adjunctive tool for BC detection, provides information on the blood flow signals, vascular supply, and corresponding hemodynamic parameters of the lesion, aiding in the evaluation of the nodule’s nature (20, 21). Malignant tumors typically show abundant vascular supply, irregular vascular morphology, and faster blood flow velocities, while benign nodules have relatively fewer (22). This study found that ultrasound characteristics of BC predominantly include hypoechoic lesions, unclear borders, irregular shape, posterior acoustic attenuation, and abnormal blood flow signals. Additionally, mammography has high *Sen* for early BC detection, especially in identifying calcifications and early lesions (23), although its limitations in detecting internal structure of masses and the associated radiation risk should still be considered (24). Research has shown that malignant BC patients typically present with fewer calcifications on pathological examination, whereas mammography often reveals irregularly shaped masses with spiculated borders and pathological calcifications (25). Another study detected 62 positive lesions in 57 BC patients, with a significantly higher proportion of lesions exhibiting irregular borders and spiculated masses (26). This study also found that, on mammography, irregular tumor borders, irregular shape, spiculated masses, calcifications, and absence of a capsule were common imaging features. Overall, MRI, ultrasound, and mammography each have unique advantages. While MRI, ultrasound, and mammography each have certain advantages in BC detection, they also have their limitations. Therefore, relying on any single method may lead to missed or incorrect diagnoses. A comprehensive evaluation of these imaging modalities can compensate for the shortcomings of individual techniques and enable more accurate diagnosis.

This study compared the diagnostic efficacy of MRI, ultrasound, mammography, and their combined methods in BC diagnosis, with a particular focus on the impact of ASSET technology in integrated diagnosis. The combined diagnostic approach in this study incorporated multiple imaging techniques, leveraging their respective strengths to enhance the *Acc* of BC diagnosis. MRI plays a crucial role in BC detection due to its high-resolution soft tissue imaging capabilities, while ultrasound offers the advantages of real-time imaging and no radiation exposure, along with the ability to assess blood flow. Mammography is widely used for early BC screening, particularly for detecting microcalcifications (27). The integration of these technologies allows for a more comprehensive acquisition of information from multiple perspectives, thereby improving the diagnostic efficacy for BC. The results of this study indicate that the combined diagnostic method significantly outperforms the use of MRI, ultrasound, or mammography alone in terms of *Acc*, *Sen*, *Spe*, and Kappa values (*P* < 0.05), which is consistent with previous findings that multimodal imaging integration helps enhance the overall diagnostic capacity for BC (28). Furthermore, the study found that MRI based on ASSET technology outperformed ultrasound and mammography in key diagnostic indicators, including *Acc*, *Sen*, *Spe*, and Kappa values. This suggests that the application of ASSET technology in MRI not only enhances scanning efficiency but also improves the visualization of lesions, thereby enhancing the diagnostic efficacy for BC. In recent years, the introduction of deep learning techniques, particularly with the aid of ASSET technology, has significantly increased MRI scanning efficiency, reduced scanning time, and minimized the impact of artifacts, leading to improved image quality (29, 30). Additionally, it has reduced scanning time while maintaining image quality (31). This study demonstrated that, compared to traditional PI technology, the ASSET-DWI sequence significantly shortened scanning time, improved lesion CNR, enhanced edge visualization, and reduced artifacts, thereby improving overall image quality (*P* < 0.05). This further validates the advantages of ASSET technology in improving imaging speed, optimizing image quality, and enhancing applicability, making it an important imaging tool in the field of MRI.

Although the advantages of ASSET combined with multimodal imaging in BC diagnosis were confirmed by this study, certain limitations remain. First, the investigation is designed as a single-center, retrospective study with a relatively small sample size; only 140 patients were enrolled (70 BC and 70 non-BC), which is insufficient to capture the diversity of clinical breast lesions and limits both statistical power and generalizability. Second, despite strict inclusion and exclusion criteria and age matching to control confounding factors, selection bias could not be entirely eliminated. In particular, the CG comprised patients with pathologically confirmed non-malignant lesions whose disease spectrum differed from that of a general screening or asymptomatic population, potentially leading to overestimation of diagnostic performance. To validate further the robustness and external value of the multimodal diagnostic strategy, a multicenter, large-sample, prospective cohort study is planned, together with the introduction of an external validation set to evaluate model generalizability across different regions and equipment configurations, thereby improving external validity and clinical applicability.

Moreover, the wider implementation of multimodal imaging-based combined diagnosis in clinical practice is still hindered by challenges in cost-effectiveness and infrastructure configuration. Although MRI, ultrasound, and mammography have already been routinely adopted in most tertiary hospitals, difficulties in equipment integration and technical coordination persists in resource-limited regions. Consequently, the strategy is considered more appropriate for refined evaluation of high-risk populations and for auxiliary diagnosis of complex cases rather than for universal screening. In the future, the establishment of standardized operating procedures and data-sharing platforms, combined with artificial intelligence-assisted interpretation, accelerated imaging sequences (e.g., ASSET), and mobile imaging devices, is expected to enhance resource utilization efficiency and system integration capacity. It promotes the sustainable application of multimodal imaging technology across broader clinical settings.

## 5 Conclusion

This study compared the acquisition time and quality of MRI images based on ASSET and PI, confirming the superiority and effectiveness of asset technology. In addition, the diagnostic performance of MRI, ultrasound, molybdenum targeted X-ray, and combined diagnostic methods was compared, and it was found that the combined diagnostic method had higher values of *Acc*, *Sen*, *Spe*, and Kappa coefficients in the diagnosis of BC. Therefore, the combination of MRI based on ASSET technology with ultrasound and mammography demonstrates potential advantages in BC diagnosis, providing more valuable imaging information for clinicians. However, since this study is not a randomized controlled trial, the results need to be further validated in larger sample sizes and multi-center studies. Additionally, the findings of this study also suggest that future research should further explore the combined use of multiple imaging techniques to enhance the *Acc* and reliability of BC diagnosis.

## Data Availability

The original contributions presented in the study are included in the article/supplementary material. Further inquiries can be directed to the corresponding author.
